# Prevalence of autism spectrum disorder in children: complete census of a Brazilian municipality

**DOI:** 10.1590/0102-311XEN255925

**Published:** 2026-09-18

**Authors:** Cassiano Forcelini, Ana Luisa Carregosa, Eduarda Leitzke, Gilmar da Silva, Regina Ampese, Itamara de Moura, Helena de Melo

**Affiliations:** 1 Universidade de Passo Fundo, Passo Fundo, Brasil.; 2 Associação de Pais e Amigos dos Excepcionais de Passo Fundo, Passo Fundo, Brasil.

**Keywords:** Autism Spectrum Disorder, Child, Censuses, Prevalence, Transtorno do Espectro Autista, Criança, Censos, Prevalência, Trastorno del Espectro Autista, Niño, Censos, Prevalencia

## Abstract

The prevalence of autism spectrum disorder (ASD) has been an issue of great concern worldwide, leading to attempts to estimate prevalence based on population samples, including those conducted in Brazil. No epidemiological study in this field has covered an entire population. We aimed to determine the prevalence of ASD in a Brazilian population. This complete census was conducted from March to December 2024 in the municipality of Coxilha, Rio Grande do Sul State, Brazil. All children aged 2.5 to 12 years were screened using the *Mini-TEA* scale. Children with a score above the 100% sensitivity threshold (> 4 points) were then examined by a neurologist with experience in ASD to establish the diagnosis according to the *Diagnostic and Statistical Manual of Mental Disorders*. Demographic data were obtained. There was no refusal to participate. Among the 475 children (240 boys and 235 girls), we found 16 ASD cases (15 boys and 1 girl), i.e., 1 in 30 children (1 in 16 boys and 1 in 235 girls), yielding an overall prevalence of 3.36% (6.25% among boys and 0.42% among girls). All ASD cases had also been diagnosed by other professionals. Only one child’s previous diagnosis of ASD was rejected. ASD showed a high prevalence and male predominance in this unprecedented complete census. The overall prevalence is similar to that reported in the United States but higher than previous Brazilian estimates. Further research is necessary to investigate etiological factors.

## Resumo

A frequência do transtorno do espectro autista (TEA) tem sido motivo de grande preocupação em todo o mundo, levando a tentativas de estimar os números com base em amostras populacionais, inclusive no Brasil. Não existe estudo epidemiológico nesta área que abranja uma população inteira. Nosso objetivo foi obter o dado de prevalência no Brasil. Este censo completo foi realizado de março a dezembro de 2024 no Município de Coxilha, Rio Grande do Sul. Todas as crianças de 2,5 a 12 anos de idade foram avaliadas com o auxílio da escala *Mini-TEA*. As crianças com pontuação acima do limiar de sensibilidade de 100% (> 4 pontos) foram então examinadas por um neurologista com experiência em TEA para definir o diagnóstico de acordo com o *Manual Diagnóstico e Estatístico de Transtornos Mentais*. Dados demográficos foram coletados. Não houve recusa em participar da pesquisa. Entre as 475 crianças (240 meninos e 235 meninas), encontramos 16 casos de TEA (15 meninos e 1 menina), ou seja, um caso de TEA para cada 30 crianças (1 para 16 meninos e 1 para 235 meninas), resultando em uma proporção de 3,36% de crianças com TEA (6,25% dos meninos e 0,42% das meninas). Todos os casos de TEA também foram diagnosticados por outros profissionais. Apenas o diagnóstico anterior de TEA de uma criança foi descartado. O TEA apresentou alta prevalência e predominância masculina neste censo completo inédito. A prevalência geral é semelhante à relatada nos Estados Unidos, porém maior do que estimativas brasileiras anteriores. Mais pesquisas são necessárias para investigar fatores etiológicos.

## Resumen

La prevalencia del trastorno del espectro autista (TEA) ha sido motivo de gran preocupación a nivel mundial, llevando a que se hayan realizado intentos por estimar las cifras a partir de muestras de la población, incluso en Brasil. No existe ningún estudio epidemiológico en este campo que abarque a toda la población. Nuestro objetivo fue obtener la cifra de prevalencia en Brasil. Este censo completo se efectuó entre marzo y diciembre de 2024 en el municipio de Coxilha, estado de Rio Grande do Sul, Brasil. Se evaluó a todos los niños de entre 2,5 y 12 años mediante la escala *Mini-TEA*. Posteriormente, los niños con una puntuación superior al umbral de sensibilidad del 100% (> 4 puntos) fueron examinados por un neurólogo con experiencia en el TEA para establecer el diagnóstico de acuerdo con el *Manual Diagnóstico y Estadístico de los Trastornos Mentales*. Se recopilaron datos demográficos. No hubo ningún caso de rechazo a participar en la encuesta. Entre los 475 niños (240 niños y 235 niñas) se identificaron 16 casos de TEA (15 niños y 1 niña), es decir, un caso de TEA por cada 30 niños (1 por cada 16 niños y 1 por cada 235 niñas), lo que da como resultado una proporción del 3,36% de niños con TEA (6,25% de los niños y 0,42% de las niñas). Todos los casos de TEA también habían sido diagnosticados por otros profesionales. Solo se rechazó el diagnóstico previo de TEA de un niño. El TEA presentó una gran prevalencia y un predominio masculino en este censo completo sin precedentes. La prevalencia general es similar a la reportada en Estados Unidos, pero más alta que las estimaciones brasileñas anteriores. Se justifica realizar más investigaciones para estudiar los factores etiológicos.

## Introduction

The diagnostic criteria of autism spectrum disorder (ASD) require persistent deficits in social communication and interaction across multiple contexts, as well as restricted, repetitive patterns of behavior, interests, or activities ^1^. These symptoms must impair social, occupational, or other important areas of functioning ^1^. No biomarkers specific to the diagnosis of ASD have been identified ^2^. The gold standard of diagnosis is the careful observation of the individual’s behavior and interviews with caregivers conducted by experienced professionals ^2^, focused on the presence of developmental abnormalities and behaviors typically first evident in infancy or childhood ^1^.

The prevalence of ASD has been an issue of great concern, leading to several attempts to estimate the true prevalence. In an incomplete census performed in Scotland in 2011 ^3^, the prevalence estimate - derived from parental reports and complemented by a projection for non-responders - was 2.9% at age 11. On the other hand, a recent meta-analysis of 79 studies worldwide found a pooled prevalence of 0.72% ^4^, but estimates were higher for studies that used records-review surveillance rather than other designs, in North America compared with other geographical regions, and in high-income compared with low-income countries. In the United States, a survey periodically reviews the registries of active surveillance programs. It extracts data from developmental evaluations and records from community medical and educational service providers in several states ^4^. The 2022 survey estimated that 1 in 31 children aged 8 years had ASD, a figure higher than previous estimates using the same method, suggesting that both the prevalence and the rate of diagnosis of ASD are increasing ^5^.

In Brazil, few studies based on questionnaires administered to mothers or caregivers aiming to estimate the prevalence of psychiatric disorders in childhood and early adolescence described symptoms suggestive of ASD in 0.3% to 0.5% of samples from Pelotas and Porto Alegre (Rio Grande do Sul State), and São Paulo ^6^ ^,^ ^7^ ^,^ ^8^. A survey conducted in Atibaia (São Paulo) using standardized instruments and clinical evaluations in a sample of children found an ASD prevalence of only 0.27% ^9^. These figures are much lower than those reported in the United States and highlight the critical need for greater research and surveillance efforts in Brazil ^10^.

Although using different approaches, all the aforementioned surveys estimated ASD frequency. Therefore, this study aimed to determine the prevalence by a complete census that assessed all children aged 2.5 to 12 years from a Brazilian municipality.

## Methods

This complete census was conducted from March to December 2024 in the municipality of Coxilha, Rio Grande do Sul, the southernmost state in Brazil. The study was approved by both the mayor of the municipality and the Research Ethics Committee of the University of Passo Fundo (approval n. 6,462,894, October 26, 2023), which was created in 2000 and is registered under number 5,342 with the Brazilian National Health Council. The survey was carried out in accordance with the *Helsinki Declaration*.

This collaborative initiative involved the UPF School of Medicine and the Association of Parents and Friends of Exceptional Children (APAE, acronym in Portuguese) of Passo Fundo. The APAE system is a nationwide network of entities devoted to the social, educational, and health care of people with disabilities. The APAE of Passo Fundo houses a Regional Reference Center for ASD of the TEAcolhe Program, a program for improving the diagnosis and management of ASD supported by the state government of Rio Grande do Sul. This survey was nested within the research portfolio of that program.

The population of Coxilha consisted of 2,667 inhabitants according to the Brazilian census conducted in 2022 ^11^. The municipality is divided into seven areas, four of which are rural. All families are visited regularly by community health workers, which allowed the creation of a municipal population registry, including those who moved in and out during the study period. According to this registry, there were 475 children aged 2.5 to 12 years in 2024. Most of them were enrolled in the two schools maintained by the municipality.

In the first phase of the survey, we invited the children’s parents (or guardians) in both schools to participate and obtained their written consent. When possible (if the child was literate), we also obtained the child’s assent. Then, a face-to-face interview with the parents/guardians was conducted to screen for ASD using the *Mini-TEA* scale, a questionnaire recently developed and validated in Brazilian Portuguese, designed for adults responsible for children aged 2.5 to 12 years (from ≥ 2.5 years to < 13 years) ^12^.

The scale yields a score ranging from 0 to 15, with 98.3% sensitivity for ASD at a cutoff score > 8. To determine the prevalence of ASD, we employed the cutoff score with 100% sensitivity (higher than 4) ^12^.

In the second phase, after screening the students, we located all children of the target age in both urban and rural areas whose parents/guardians had not been interviewed at the schools. This was accomplished with the help of the community health workers who were regularly updating the municipal population registry, resulting in complete coverage of the target population. There was no refusal to participate in the survey.

In parallel with the first and second screening phases, all children whose parents/guardians scored above 4 on the *Mini-TEA* scale were evaluated by a single neurologist with experience in neurodevelopmental disorders according to the criteria of the *Diagnostic and Statistical Manual of Mental Disorders, 5th Edition, Text Revision* (DSM-5-TR) ^1^.

Demographic data from all 475 children included age, sex, and area of origin (urban or rural). We recorded the diagnoses of ASD and other neurodevelopmental disorders established during the evaluation of children whose parents/guardians scored above 4 on the *Mini-TEA* scale. The time spent interviewing parents/guardians during screening was also recorded.

Categorical variables are presented as absolute numbers and percentages; quantitative data are described with median and interquartile range (IQR) because of asymmetric distribution. Only descriptive statistics were performed to describe the study population as this survey is a complete census and does not require inferential statistics derived from samples ^13^.

## Results

**Figure 1:**
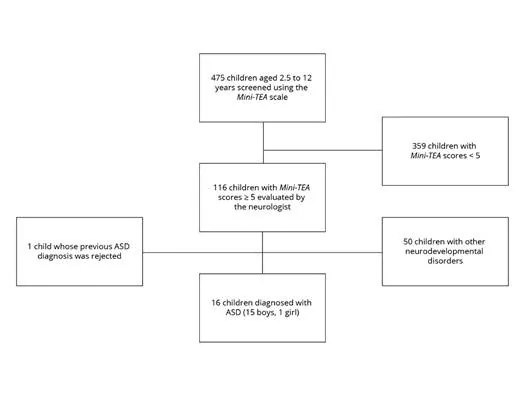
Study flowchart from screening of the target population to diagnostic confirmation of autism spectrum disorder (ASD) cases.

Figure 1 shows the study flowchart. Among the 475 children aged 2.5 to 12 years (240 boys and 235 girls), we identified 16 ASD cases (15 boys and 1 girl). This corresponds to 1 in 30 children (1 in 16 boys and 1 in 235 girls), i.e., an overall prevalence of 3.36% (6.25% of boys and 0.42% of girls).

The children diagnosed with ASD had already been diagnosed or were diagnosed during the research period by other professionals (neurologists or psychiatrists). Notably, the study neurologist rejected only one child’s previous ASD diagnosis because the child did not meet the diagnostic criteria.

**Figure 2:**
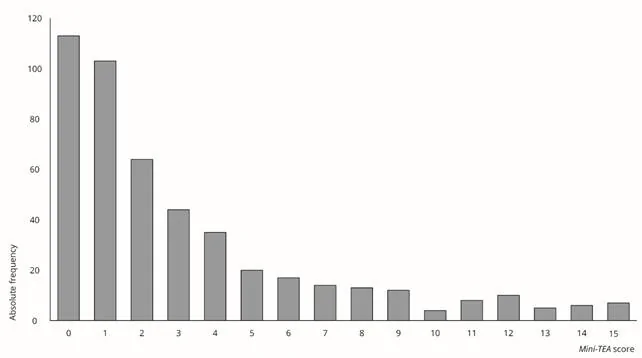
Distribution of *Mini-TEA* scores across the study population (n = 475).

**Figure 3:**
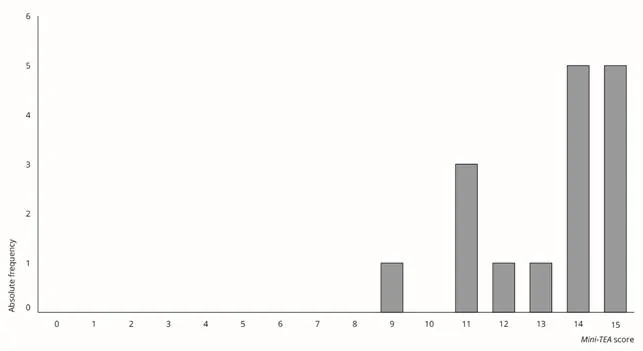
*Mini-TEA* scores among children with autism spectrum disorder (ASD) (n = 16).

Table 1 presents the demographic characteristics of the total population and of the ASD and non-ASD groups. The distribution of *Mini-TEA* scores across the population is shown in Figure 2, and the scores for the ASD cases are shown in Figure 3. Scores for the ASD cases ranged from 9 to 15, with most scores being 14 or 15. The median time spent administering the screening scale to parents/guardians was 7.1 minutes (IQR: 6-9.5 minutes).

**Table 1:** Clinical and sociodemographic characteristics of the sample.

Characteristics	General population (n = 475)	ASD (n = 16)	Non-ASD (n = 459)
Age (years)			
Median	7.7	5.1	7.8
IQR	4.9-9.9	3.5-9.7	4.9-9.9
*Mini-TEA* score			
Median	2	14	2
IQR	1-4	11-15	1-4
Sex			
Male	240	15	225
Female	235	1	234
Area of origin			
Urban	343	15	328
Rural	132	1	131

ASD: autism spectrum disorder; IQR: interquartile range.

Table 2 describes the frequency of neurodevelopmental disorders among children who scored above 4 on the *Mini-TEA* scale (116 subjects, i.e., 24.4% of the study population). All children with such a diagnosis or with symptoms consistent with other clinical or psychiatric conditions were referred for neurological, psychiatric, or pediatric follow-up as appropriate.

**Table 2:** Frequency of neurodevelopmental disorders (n = 66) among children who scored above 4 on the *Mini-TEA* scale (n = 116). Diagnoses were established by the neurologist according to *Diagnostic and Statistical Manual of Mental Disorders, 5th Edition, Text Revision* (DSM-5-TR) criteria.

Diagnosis	n
ASD	16
ADHD	26
Intellectual disability	8
Intellectual disability + ADHD	6
ODD + ADHD	6
Global developmental delay	2
Language disorder	2

ADHD: attention-deficit/hyperactivity disorder; ASD: autism spectrum disorder; ODD: oppositional defiant disorder.

## Discussion

This is the first survey to evaluate an entire population for ASD. We screened all children of the target age and carefully examined those with *Mini-TEA* scores above the threshold corresponding to 100% sensitivity, without any refusal - an unprecedented complete census.

Our findings show a high prevalence of ASD, which we partly attribute to the complete population coverage. The prevalence estimate is indeed very close to that published in the most recent bulletin of the U.S. Autism and Developmental Disabilities Monitoring (ADDM) Network ^5^. The ADDM Network surveillance, conducted periodically in the United States, is considered the most robust epidemiological survey, even though it does not directly evaluate the children. The convergence of results, despite different approaches, locations, and populations, supports the observed prevalence estimate.

Previous studies on the prevalence of symptoms suggestive of ASD in a few Brazilian cities found figures much lower than those reported in our survey ^6^ ^,^ ^7^ ^,^ ^8^ ^,^ ^9^, probably because of methodological limitations, including the sampling method ^10^. The Brazilian Institute of Geography and Statistics (IBGE, acronym in Portuguese), the Brazilian agency responsible for the national census, recently published a note indicating an estimate of the frequency of ASD in the population according to age, derived from the incomplete census conducted in 2022 ^14^. The highest figure was 2.6% (1 in 38) among children aged 5 to 9 years. We believe this is an underestimate due to the sampling methods: census agents did not visit all households; only a proportion of respondents were randomly asked about an ASD diagnosis among household members; and those reported to have ASD were not clinically evaluated by the census agents. A similar methodology was used in Scotland in 2011 and yielded an estimated prevalence of 2.9% at age 11, with the same limitations ^3^.

This epidemiological survey reinforces the value of the *Mini-TEA* as a screening method for ASD in Brazilian Portuguese and its clinical cut-off score (≥ 9) with high sensitivity ^12^. Most children had a low score, whereas all cases of ASD scored from 9 to 15. In a context where the *Modified Checklist for Autism in Toddlers, Revised with Follow-up* (M-CHAT-R/F) is not widely used for children under 2.5 years, despite the availability of a translated and validated form ^15^ ^,^ ^16^, the *Mini-TEA* scale provides a second opportunity for those who were not screened previously. A theoretical limitation of the survey is the absence of diagnostic evaluation in children who scored below the screening cut-off score (< 5 points) on the *Mini-TEA* scale, potentially resulting in false-negative cases. However, the scale was validated in two studies involving hundreds of children evaluated for fulfillment of DSM-5-TR criteria ^12^ ^,^ ^17^, a large proportion of whom had ASD. The cut-off score used in this survey had 100% sensitivity in those studies.

ASD was not the only diagnosis associated with moderate or high *Mini-TEA* scores, an expected finding given the scale’s moderate specificity (59% at a cut-off ≤ 8). This explains the 50 cases of other neurodevelopmental disorders diagnosed among the 116 children who scored above 4.

The DSM-5-TR criteria were chosen because of logistical and economic constraints. Established diagnostic instruments such as the *Autism Diagnostic Observation Schedule, 2nd Edition* (ADOS-2), and the *Autism Diagnostic Interview - Revised* (ADI-R) are expensive and time-consuming, making them infeasible for an extensive census. This should be recognized as a limitation, particularly because the comprehensive clinical evaluation such tools can provide was not obtained. Another limitation is the lack of a second rater for diagnostic confirmation; all diagnoses were made by a single neurologist, albeit one with expertise in neurodevelopmental disorders, which raises the possibility of measurement bias. However, all children diagnosed with ASD by the study neurologist had been diagnosed previously or during the research period by other attending neurologists.

Although ASD is highly familial, indicating a strong genetic contribution, epigenetic and environmental factors may also play important roles ^18^. This complexity makes identifying possible etiologic factors challenging. The absence of an etiological investigation in our survey is a limitation, but the study was not designed for that purpose.

Male predominance is a ubiquitous finding worldwide ^5^ ^,^ ^19^ ^,^ ^20^, but our male-to-female ratio was the highest ever reported. This inevitably raises concern, reflecting a current issue in the literature: the possibility of underdiagnosis among females ^21^ ^,^ ^22^ ^,^ ^23^. Many girls with ASD exhibit mild symptoms and may escape early diagnosis, being identified only in adulthood, if ever ^21^. In addition, the use of ADOS as a confirmatory diagnostic measure has been linked to the exclusion of females with ASD from the research studies at a rate over 2.5 times higher than that of males with ASD ^22^. This concern does not apply to our survey because we did not use ADOS. Nonetheless, the possibility of missing a girl with extremely mild ASD cannot be completely excluded, although we consider it remote because we screened all children from the community with a high sensitivity instrument and evaluated all those above the cut-off. Resolving this issue requires further census-based studies to avoid sampling bias and to determine the true prevalence of ASD.

An additional limitation is that Coxilha is a small municipality with a predominantly agricultural economy and a significant proportion of inhabitants living in rural areas, a reality far different from that of medium-sized and large cities. This may limit the generalization of our results to the Brazilian population. Nevertheless, this pioneer initiative serves as a starting point to encourage further epidemiological research in Brazil. Sampling strategies in different settings will be necessary, given the logistical obstacles to conducting a complete census in a large urban population. We emphasize the importance of this study for planning the health resources required to adequately support people with ASD in Brazil.

In conclusion, this unprecedented complete census screened children in a Brazilian municipality for ASD using the *Mini-TEA* scale. The results revealed the highest prevalence and male-to-female ratio ever reported. The prevalence estimate is close to that reported in the United States and higher than those described in previous Brazilian studies that assessed symptoms suggestive of ASD or ASD diagnoses in population samples, including the last national census. Because of the complete population coverage, we propose the city of Coxilha as a promising site for further etiologic research. Finally, these results should guide public health policies and help private health insurers provide adequate care for people with ASD.

## Data Availability

The research data are available upon request to the corresponding author.
